# Exploring the link between ultraviolet B radiation and immune function in amphibians: implications for emerging infectious diseases

**DOI:** 10.1093/conphys/coy035

**Published:** 2018-06-28

**Authors:** Rebecca L Cramp, Craig E Franklin

**Affiliations:** School of Biological Sciences, The University of Queensland, Goddard Building (8), St Lucia, Queensland, Australia

**Keywords:** adaptive, *Batrachochytrium dendrobatidis*, chytridiomycosis, embryo, innate, larvae

## Abstract

Amphibian populations the world over are under threat of extinction, with as many as 40% of assessed species listed as threatened under IUCN Red List criteria (a significantly higher proportion than other vertebrate group). Amongst the key threats to amphibian species is the emergence of novel infectious diseases, which have been implicated in the catastrophic amphibian population declines and extinctions seen in many parts of the world. The recent emergence of these diseases coincides with increased ambient levels of ultraviolet B radiation (UVBR) due to anthropogenic thinning of the Earth’s protective ozone layer, raising questions about potential interactions between UVBR exposure and disease in amphibians. While reasonably well documented in other vertebrate groups (particularly mammals), the immunosuppressive capacity of UVBR and the potential for it to influence disease outcomes has been largely overlooked in amphibians. Herein, we review the evidence for UVBR-associated immune system disruption in amphibians and identify a number of direct and indirect pathways through which UVBR may influence immune function and disease susceptibility in amphibians. By exploring the physiological mechanisms through which UVBR may affect host immune function, we demonstrate how ambient UVBR could increase amphibian susceptibility to disease. We conclude by discussing the potential implications of elevated UVBR for inter and intraspecific differences in disease dynamics and discuss how future research in this field may be directed to improve our understanding of the role that UVBR plays in amphibian immune function.

## Teaser

Levels of ultraviolet B radiation (UVBR), a potent immunosuppressant, have increased in parts of the world due to anthropogenic thinning of the Earth’s protective ozone layer. However, little is known about how UVBR influences amphibian immune function, or if increased UVBR exposure can contribute to disease emergence in susceptible species.

## Introduction

Amphibians are currently the vertebrate taxon most threatened with extinction (Houlahan *et al.*, 2000; Hof *et al.*, 2011; IUCN, 2016). Global amphibian numbers have undergone substantial declines over the last four decades, with between 40 and 50% of examined species listed as at least ‘Near Threatened’ by the International Union for the Conservation of Nature (IUCN) Red List of Threatened Species (IUCN, 2016). A major contributor to recent amphibian declines was determined following the discovery of the novel fungal pathogens *Batrachochytrium dendrobatidis* (*Bd*) in 1998 (Berger *et al.*, 1998) and *Batrachochytrium salamandrivorans* (*BSal*) (Martel *et al.*, 2013). Both pathogens cause the deadly amphibian disease chytridiomycosis (Berger *et al.*, 1998; Longcore *et al.*, 1999). Since its identification almost 20 years ago, *Bd* has been recorded in over 500 amphibian species worldwide and has been implicated in the decline or extinction of several hundred of these (Berger *et al.*, 1998; Lips *et al.*, 2006; Cheng *et al.*, 2011; Van Rooij *et al.*, 2015). In addition to *Bd* and *Bsal*, several other diseases caused by amphibian pathogens (Ranaviruses and trematode parasites) have recently increased in prevalence. Ranaviruses have now been detected in more than 70 amphibian species in 20 countries (Miller *et al.*, 2011; Gray and Chinchar, 2015), making them the second most common infectious agent of amphibians after *Bd* (Chen and Robert, 2011). Similarly, trematode infections caused by the parasite *Riberioa ondatrae* have increased in North American amphibians to such an extent that they have contributed significantly to recent local population declines (reviewed by Rohr *et al.*, 2009).

An apparent rise in the number of disease-related amphibian declines has prompted questions as to why such a trend should be occurring now (Hof *et al.*, 2011; Blaustein *et al.*, 2012; James *et al.*, 2015; Kolby and Daszak, 2016). Increased contact between wildlife, humans and domestic animals has undoubtedly amplified the exposure of wildlife to novel, potentially pathogenic organisms (Daszak *et al.*, 2000). However, the consequences of a novel host–pathogen interaction depend not only on the specific physical/physiological characteristics of the host and pathogen, but also on how these are shaped by their interactions with the surrounding environment (Daszak *et al.*, 2001; Blaustein *et al.*, 2012). Environmental conditions not only affect pathogen growth, transmissibility and pathogenicity but also influence host behaviour, immune function and pathogen exposure regimes (Fisman, 2007). Host immune function is sensitive to a range of biotic and abiotic environmental variables like temperature (Engelsma *et al.*, 2003; Raffel *et al.*, 2006; Ndong *et al.*, 2007; Barber *et al.*, 2016), habitat quality (Cary *et al.*, 2014; Katzenback *et al.*, 2014; Krynak *et al.*, 2015; Makrinos and Bowden, 2016), nutritional status (Venesky *et al.*, 2012), competition (Groner *et al.*, 2014) and importantly, solar UVB radiation (Kripke *et al.*, 1992; Lahnsteiner *et al.*, 2011; Debecker *et al.*, 2015; Abu Bakar *et al.*, 2016).

Ultraviolet B radiation radiation (UVBR) forms a part of the solar electromagnetic spectrum (wavelength range 280–320 nm). Although the majority of solar UVBR reaching the outer atmosphere is absorbed by stratospheric ozone, a small amount does reach the Earth’s surface (van der Leun, 2004). Spatial and temporal variations in ozone thickness influence UVBR levels at the Earth’s surface. Other factors, such as solar angle and proximity, cloud cover, surface reflectance (albedo) and altitude, also influence terrestrial UVBR levels (Xenopoulos and Schindler, 2001). While UVBR can penetrate into aquatic environments, surface waves, reflectivity and levels of dissolved organic carbon (DOC) significantly affect the transmission of UVBR and the depth to which it can influence aquatic systems (Xenopoulos and Schindler, 2001).

UVBR is a powerful natural stressor because it can interact with a range of biological molecules and is capable of causing extensive cellular and molecular (DNA and protein) damage (Diffey, 1991). At the organismal level, UVBR exposure can adversely impact survival, growth rates, developmental trajectories, locomotion and predator avoidance. In amphibians, embryonic and larval stages are particularly sensitive to UVBR. These life stages are at increased risk from UVBR as they are often exposed to direct sunlight, are most commonly found during spring and summer (when UV levels are highest) and/or have a limited capacity to avoid UVBR exposure (Blaustein and Belden, 2003). UVBR can have a variety of effects on embryonic and larval amphibians including increased mortality (e.g. Crump *et al.*, 1999; Belden *et al.*, 2003; Alton *et al.*, 2010), decreased growth and impaired development (e.g. Belden and Blaustein, 2002; Calfee *et al.*, 2006), developmental abnormalities (e.g. van Uitregt *et al.*, 2007; Romansic *et al.*, 2009) and reduced locomotor performance and altered behaviour (e.g. Kats *et al.*, 2000; van Uitregt *et al.*, 2007). UVBR can also modulate the effects of other stressors (both biotic and abiotic) on amphibians (reviewed by Alton and Franklin, 2017). However, not all species respond to UVBR in the same way (Licht and Grant, 1997). Responses to UVBR can vary with geographic and seasonal exposure patterns (Peterson *et al.*, 2002), as well as with the capacity to avoid and/or repair DNA damage (e.g. Hansen *et al.*, 2002; Blaustein *et al.*, 2004; Palen *et al.*, 2005; Thurman *et al.*, 2014).

Studies on non-amphibian taxa show UVBR is both genotoxic and a powerful modulator of immune function, with studies of fish and mammal species showing increased incidence of, and susceptibility to, disease when exposed to sublethal doses (Salo *et al.*, 2000; de Gruijl, 2008; Blount and Pike, 2012; Siroski *et al.*, 2012; Ullrich and Byrne, 2012). In comparison with other vertebrate taxa, the impacts of UVBR on amphibian immune function and disease are poorly documented and the role of UVBR in disease-related mortality and decline of amphibian populations remains conjectural. There are, however, a number of lines of evidence to suggest that disease-related amphibian declines in some areas may be linked to increased UVBR exposure. For example, many *Bd*-related amphibian declines have occurred in montane environments (Lips, 1998; Middleton *et al.*, 2001; Bosch *et al.*, 2007; Kriger and Hero, 2008; Rohr and Raffel, 2010; Walker *et al.*, 2010) where ambient UVBR levels are significantly higher than at lower altitudes because there is less atmospheric filtration of UVBR (UV levels increase by 10–12% for every 1000 m of elevation) (Madronich *et al.*, 1998). In addition, many declines attributable to disease (e.g. declines in eastern Australia and South America during the late 1970s and 1980s) coincide spatially and/or temporally with increases in UVBR associated with stratospheric ozone depletion (Berger *et al.*, 1998; Lips, 1998; Lips *et al.*, 2006).

UVBR-induced immunosuppression was suggested as a potential factor contributing to amphibian declines as far back as 1993 (Carey, 1993). However, the impacts of UVBR exposure on amphibian immune function and disease susceptibility have been little studied since, and much remains unknown about whether changes in global UVBR levels have contributed to recent disease-associated amphibian declines. In the relatively small number of studies that have attempted to address this issue, results have been conflicting and/or inconclusive. For example, Garcia *et al.* (2006) found that exposure of juvenile (post-metamorphic) frogs of three species (*Rana cascadae*, *Bufo boreas* and *Hyla regilla)* to low levels of UVBR for 3 days did not increase susceptibility to *Bd* or subsequent mortality. Likewise, Searle *et al.* (2010) found no increase in mortality rates or in the infectiousness of *Bd* when *Rana cascade* larvae were exposed to UVBR and *Bd* simultaneously. UVBR exposure has also been linked to reduced *Bd* susceptibility in larvae of *Bufo bufo* (Ortiz-Santaliestra *et al.*, 2011) but increased *Bd* susceptibility in green tree frog (*Litoria caerulea*) larvae (Cramp and Franklin, preliminary unpublished observations). Differences in the source of the UVBR (natural or artificial), UVBR dose, the timeframe of exposure, life history stage at the time of exposure and species influence amphibian UVBR exposure outcomes (Licht and Grant, 1997; Alton and Franklin, 2017). Experimental differences across existing studies may also contribute to the lack of consensus on the role that UVBR might play in *Bd* susceptibility. Although *Bd* epidemiology is undeniably complex, the links between chytridiomycosis and elevation/altitude, with increased prevalence/severity of chytridiomycosis at higher altitude sites, are clear. With UVBR levels increasing with altitude (Madronich *et al.*, 1998), it is conceivable that the greater prevalence of this and other diseases at high altitude may be related to increased UVBR exposure. Currently, our capacity to understand the relationship between UVBR and disease outcomes is significantly hampered by the lack of understanding of the fundamental way(s) in which UVBR can interact with, or modulate, amphibian immune function.

## Pathways to impact: mechanisms through which UVBR can influence amphibian immune function

The vertebrate immune system is a suite of complex, interrelated morphological, physiological, cellular and chemical facets that animals use to determine self from non-self (Schulenburg *et al.*, 2009). Immune defences can be classified as innate or adaptive; innate immune defences are considered the ‘first line of defence’ and provide rapid, non-specific protection against a variety of potential pathogens. The innate immune system includes physical barriers like the skin which impedes the movement of microbes into the body, mucus which often contains a variety of antimicrobial substances and the hosts’ own commensal microbiome with which a potential pathogen must successfully outcompete in order to become an established infection. The innate immune system also includes cell-mediated defences in the form of white blood cells and chemical cascades like complement and lysozyme. The acquired or adaptive immune system is a set of immune responses highly specific to the pathogen that induced them and includes the production of antibodies against foreign antigens and the retention of immunological memory. The functions of the vertebrate immune system can be affected by exposure to a range of environmental factors and through the effects of other (competing) physiological processes.

The amphibian immune system is similar to that of most other vertebrates in that it incorporates both innate and adaptive immune pathways (Carey *et al.*, 1999). In amphibians, as in other vertebrate taxa, the immune defences of early life (embryonic and larval) stages are less well developed than those of adult frogs. Some embryonic immune defences are, nevertheless, established very early (Rollins-Smith, 1998) with egg membranes forming a direct barrier against pathogen entry (Robert and Ohta, 2009). There is also some evidence that maternal antibodies are passed into the eggs during vitellogeneis providing additional protection during embryonic development (Poorten and Kuhn, 2009). Initial adaptive immunological competence is achieved approximately 2 weeks post-fertilization in *Xenopus laevis* larvae with the development of the principle lymphoid tissues, the spleen and thymus (Robert and Ohta, 2009). Larval adaptive immune function is comparatively less robust than that of adult amphibians, with larval amphibians having fewer lymphocytes and reduced antibody diversity than adults (Rollins-Smith, 2017). During metamorphosis, hormonal changes result in a sharp reduction in larval lymphocyte abundance (immunosuppression) as the immune system is reorganized from the larval type to the adult type to prevent the larval immune system from attacking the newly formed adult tissues (Rollins-Smith, 1998; Robert and Ohta, 2009). Following metamorphosis, immune function is gradually restored, however, mature immune function may not be achieved for up to a year post-metamorphosis (Rollins-Smith, 1998, 2017). Taken together, the larval and juvenile life history stages are comparatively most at risk from disease or infection because of the immaturity of the immune system. It is not surprising then that many amphibian diseases are most problematic for embryonic, larval and juvenile life stages (Gray *et al.*, 2009; Miller *et al.*, 2011; Langhammer *et al.*, 2014).

**Figure 1:**
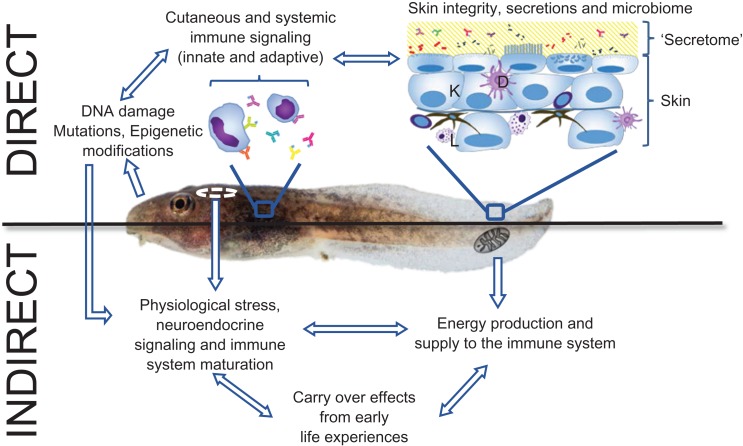
Schematic representation of the potential direct and indirect pathways through which UVBR can influence immune function in amphibian early life stages. UVBR can directly kill and damage cells in the outer skin layers, disrupting the physical integrity of the skin, compromising the function of cutaneous dendritic cells (D) and leucocytes (L), and triggering exposed keratinocytes (K) to release a cascade of immunosuppressive molecules that inhibit innate and adaptive immune functions of the systemic immune system. UVBR can disrupt the innate immune function of the cutaneous ‘secretome’ (mucus, antimicrobial peptides, complement, lysozyme, etc.) and influence the composition of the host microbiome. Indirectly, UVBR may influence immune function via its impact on interrelated physiological systems. For example, UVBR can affect immune function by disrupting energy production and/or distribution pathways, by influencing neuroendocrine signalling pathways controlling immune system maturation or inducing a physiological stress response (involving glucocorticoids). UVRB-associated damage to DNA and other biomolecules may also have a lasting impact on immune function by influencing gene expression patterns in subsequent life stages. Image: Cameron Baker©.

Embryonic and larval amphibians are more likely to be exposed to UVBR than adult stages, although several declining anuran and urodele species (i.e. *Taudactylus eungellensis and Taudactylus acutirostris*, several *Atelopus spp*. and a number of aquatic salamander species) also have diurnal juvenile and adult stages for which UVBR exposure may be harmful. In the subsequent sections of this review, we will explore how UVBR exposure influences immune defences early in development (embryonic, larval and juvenile). We will show that UVBR can act directly on components of the innate and adaptive immune systems, as well as indirectly influencing immune function through impacts on other physiological systems or by influencing the capacity of other environmental stressors to affect immune function (Figure 1). In doing so, we review not only studies examining the impacts of UVBR on amphibian species directly but also relevant studies on other taxa where data for amphibians is lacking. In reviewing the evidence for potential effects of UVBR on amphibian immune function, we also identify gaps in our understanding of how UVBR, through its impact on immune function, may contribute to disease processes in amphibians.

### Direct modulation of immune system function by UVBR

#### Skin integrity

The barrier function of epithelia is an integral component of the innate immune system of all animals and serves as the ‘first line of defence’ against potential microbial invasion. Damage to the integrity of the skin compromises the barrier role of the skin and increases the capacity for pathogens to enter the tissues and cause infection. In most vertebrates, UVBR is absorbed by the epidermis and often does not penetrate into the deeper skin layers (Biniek *et al.*, 2012). However, larval amphibian skin is potentially highly sensitive to UVBR, since their protective pigment (melanin) layers lie largely beneath the epidermis within the dermis (Fox, 1986). In most taxa, including amphibians, acute responses to elevated UVBR can include sunburn (Blazer *et al.*, 1997; Little and Fabacher, 2003), melanogenesis (skin darkening), lesions on the dorsal skin and eyes (McFadzen *et al.*, 2000; Zamzow, 2004; Kazerouni and Khodabandeh, 2010), necrosis and sloughing of the skin (Noceda *et al.*, 1997; Abedi *et al.*, 2015), a reduction in epidermal strength (Biniek *et al.*, 2012), and epidermal hyperplasia and oedema (Matsumura and Ananthaswamy, 2004). Larvae of two frog species, *H. regilla* and *Rana aurora*, experienced both skin damage and lens opacity following exposure to elevated levels of artificial UVBR (Flamarique *et al.*, 2000). Subsequent fungal infections in areas of UV-related skin damage have been reported (Flamarique *et al.*, 2000; Little and Fabacher, 2003), indicating that UVBR exposure can compromise the immunological barrier function of amphibian skin. Whether these results apply to natural exposure regimes is presently unclear as the aforementioned studies were undertaken under laboratory conditions using artificial light sources.

While exposure to elevated UVBR can sometimes lead to obvious outward signs of skin damage, sub-erythemal exposures can also lead to levels of barrier dysfunction that can precede infection by secondary fungal pathogens (Nowak, 1999). Since sub-erythemal skin damage is difficult to determine macroscopically, it may go unnoticed in wild amphibian studies where levels of embryonic and larval mortality are naturally very high anyway. If, like in other taxa, sub-erythemal skin damage increases the risk of pathogen infections in early amphibian life stages, further work is needed to better understand how UVBR exposure regimes influence infection outcomes. Moreover, exposure ‘thresholds’ are likely to vary across species and populations with the capacity of animals to behaviourally and/or morphologically limit their exposure to, or tolerate, UVBR in their environment. Similarly, since UVBR levels vary temporally and spatially, exposure regimes (fluctuating vs acute exposures) may also affect skin responses within and across life history stages. We, therefore, need to examine the possibility that early UVBR-associated skin damage may have long-term consequences for cutaneous immune function in later life history stages and appreciate that the acute and long-term effects of UVBR on skin function are likely to vary both between and within species.

#### Skin secretions

Skin secretions form a vital component of the innate immune system of all animals. Secretions function as both a physical barrier to pathogens, by trapping and preventing their establishment on the skin surface, and as a chemical barrier, containing a variety of antimicrobial proteins and peptides like proteases, lectins and lysozyme, which actively kill microbes on contact (Ellis, 2001; Zasloff, 2002; Magnadottir, 2006). Embryonic and larval amphibians have several types mucus- and peptide-secreting cells in the integument (Altig and McDiarmid, 1999) that produce substances with well-defined immunoprotective functions (Woodhams *et al.*, 2016). Damage to this layer can directly increase the risk of pathogen infection (Kanno *et al.*, 1989; Magarinos *et al.*, 1995; Zamzow and Siebeck, 2006). In some reef fish, cutaneous mucus also (indirectly) influences the innate immune defences (i.e. the barrier function of skin) by reducing the penetrance of UVBR through to the more UVBR-sensitive skin layers (Zamzow and Losey, 2002; Zamzow, 2004; Zamzow and Siebeck, 2006; Eckes *et al.*, 2008); whether larval amphibian mucus has a similar photoprotective function is largely unknown. Notably, UVBR exposure can reduce the abundance and distribution of mucus-secreting cells in fish larvae (Kaweewat and Hofer, 1997; McFadzen *et al.*, 2000; Kazerouni and Khodabandeh, 2010; Abedi *et al.*, 2015). It is currently uncertain if UVBR exposure can influence mucus production in amphibians. The loss of mucus-producing cells may be particularly problematic for embryonic and larval animals whose adaptive immune systems are underdeveloped relative to adult frogs (Rollins-Smith, 1998).

Non-mucus skin secretions are an important component of the innate immune system of larval, juvenile and adult amphibians (Woodhams *et al.*, 2007, 2016). Antimicrobial peptides (AMPs) have been shown to be a particularly important constituent of non-mucus skin secretions, since many are effective against cutaneous pathogens like *Bd* (Rollins-Smith, 2009). Indeed, the diversity and abundance of cutaneous AMPs is thought to be a major factor contributing to the differential susceptibility of frog species to *Bd* (Rollins-Smith, 2009). Abiotic stressors such as pollutants and low environmental temperatures can differentially influence the production, composition and release of AMPs from skin glands (Davidson *et al.*, 2007). However, little is known about the capacity for UVBR exposure to directly influence the production and/or composition of cutaneous AMP in larval amphibians or for early UVBR exposures to affect AMP production or composition in subsequent life history stages.

As far as we could determine, no studies have explored the hypothesis that early UVBR exposure can disrupt the physiochemical properties of embryonic and larval amphibian skin. Laboratory studies are required to establish a cause-and-effect relationship between UVBR exposure and skin secretion properties. If such a relationship can be established, it will be important to ascertain how the timing, dose and intensity of UVBR exposure might affect the subsequent composition of the cutaneous ‘secretome’ or influence the subsequent development of cutaneous mucus and glandular tissues.

#### The cutaneous microbiome

The microbiome of the amphibian integument plays an important role in host immunity and disease progression (Longo *et al.*, 2015; Rebollar *et al.*, 2016). As with all organisms, the ‘external’ surfaces of amphibians are colonized by a large and diverse array of microbiota (bacteria, fungi, viruses and mites), most of which are commensal or transient and cause no harm at all to the host. A subset of these microbes produce metabolites that actively discourage establishment of potential pathogens, thereby contributing to disease resistance (Patra *et al.*, 2016). The host’s microbiome also provides a highly competitive microenvironment in which a potential pathogen must outcompete the commensal microbiota in order to become established. The composition of the host microbiome can determine the outcome of infection by pathogens, with recent studies showing variation in host susceptibility to *Bd* is linked to the presence/absence of certain bacteria on the host’s integument (Briggs *et al.*, 2010; Jani *et al.*, 2017).

Given the links between *Bd* infection outcomes and microbiome composition, there is considerable interest in understanding those factors that may regulate or disturb amphibian microbiomes. Like all ecosystems, host-associated microbiomes are sensitive to environmental disturbances. Aquatic pH, salinity and temperature have been identified as primary regulators of host- and non-host-associated microbial communities (Fierer and Jackson, 2006; Lozupone and Knight, 2007; Costello *et al.*, 2009; Kueneman *et al.*, 2014). Solar UVBR has well-documented antimicrobial properties, capable of affecting both commensal and pathogenic organisms (Faergemann and Larko, 1987; Dotterud *et al.*, 2008; Wang *et al.*, 2012). Recent work in humans suggests that UVB radiation can modulate aspects of the host microbiomes more broadly, and in doing so, influence disease development and outcomes (Patra *et al.*, 2016). However, the role that UVBR may play in the regulation of innate immune function in amphibians, through its impact on host microbiome composition remains largely unknown. In the only study to date that has addressed this question in amphibians, pond shading had no impact on larval microbiome composition in *Rana catesbeiana* (Krynak *et al.*, 2015), suggesting that UVBR may not influence the structure of the microbiome in some aquatic amphibians. However, since UVBR penetrance into aquatic systems is dependent on the amount of dissolved organic matter in the water, pond shading levels and solar angle (Xenopoulos and Schindler, 2001), further work is needed to determine if aquatic microbiomes can actually be influenced by natural levels of UVBR in other environs where UVBR penetrance is higher (e.g. at high altitude, clear stream sites).

#### Cutaneous and systemic immune signalling

The cutaneous immune system comprises interconnected innate and adaptive cellular networks involved in localized and systemic immune responses to potential pathogens (Mann *et al.*, 2012). In vertebrate skin, keratinocytes in the epidermis produce a complex array of AMPs, pro-inflammatory cytokines and chemokines in response to the activation of pathogen recognition receptors on their cells’ surfaces (Mann *et al.*, 2012). In addition to these cells, antigen-presenting dendritic cells (Langerhans cells) actively patrol the integument for would-be pathogens. These cells perform a vital role bridging the cutaneous innate and adaptive immune systems: they can recognize a diverse array of pathogen- and self-associated molecules and can initiate the appropriate immune or tolerogenic responses (Sleijffers et al., 2004; Mann et al., 2012).

The effects of UVBR exposure on cutaneous immune signalling in amphibians is largely unexplored; however, data from mammalian models show that UVBR exposure can induce local and systemic immunosuppression by reducing cutaneous dentritic cell abundance, and by reducing their capacity to recognize antigens and induce an adaptive immune response (Kripke, 2013). In addition, UVBR exposure induces the secretion of immunosuppressive cytokines (including interleukin-10) by keratinocytes, which suppresses other local and systemic immune responses (Elmets *et al.*, 1983; Beissert and Schwarz, 1999) and impedes DNA damage repair mechanisms (Sreevidya *et al.*, 2008). While data relating to non-mammalian taxa are limited, studies on fish suggest a common action of UVBR on elements of the systemic immune system. In some fish species, UVBR exposure correlates with a reduction in the abundance of circulating peripheral lymphocytes (Jokinen *et al.*, 2000, 2001; Markkula *et al.*, 2005, 2007, 2009), with a reduced functional capacity of white blood cells to respond to stimulation (Salo *et al.*, 1998; Markkula *et al.*, 2009) and with lower blood immunoglobulin levels (Jokinen *et al.*, 2001, 2008, 2011; Markkula *et al.*, 2005).

Importantly, local and systemic immunomodulation by UVBR exposure has been directly linked to a wide range of mammalian pathogen infection outcomes (reviewed by Sleijffers *et al.*, 2004). Similarly in fish, UVBR can negatively affect pathogen infection rates even when UVBR doses are not sufficient to cause overt skin damage (Markkula *et al.*, 2007; Cramp *et al.*, 2014). Moreover, the effects of UVBR on infection susceptibility can manifest after just a single exposure and can persist for several weeks (Jeevan and Kripke, 1989). The effects of UVBR on cutaneous immune function and systemic signalling in amphibians (larval or adult) are largely unknown. However, given the similarly of the amphibian immune system to that of other vertebrates (Carey *et al.*, 1999), it is not unreasonable to expect that similar responses to UVBR might occur.

### Indirect modulation of immune function by UVBR

#### Physiological stress, neuroendocrine signalling and immune system maturation

UVBR can exert its influence on immune function through its capacity to influence a wide range of endocrine functions. Hormones mediate many of the mechanisms that underpin phenotypic responses to environmental stimuli (Gilbert and Epel, 2008); in particular, hormones of the systemic stress axis (hypothalamic–pituitary–adrenal in mammals and birds, or hypothalamic–pituitary–intrarenal axis in fish, amphibians and reptiles) are principle mediators of physiological responses to environmental stimuli (Denver, 2009). These hormones include glucocorticoids (corticosterone and cortisol) released from the adrenal (intrarenal) tissues during stressful events, which function to rapidly mobilize energy supplies by reducing the energy supplied to non-vital physiological processes. While beneficial in the short-term, chronically elevated glucocorticoid levels can be maladaptive, suppressing immune function and increasing susceptibility to disease (Wedemeyer, 1970; Snieszko, 1974; Barton and Iwama, 1991; Engelsma *et al.*, 2003; Murray and Peeler, 2005; Bowden, 2008; Tort, 2011). Chronically elevated glucocorticoid levels may increase pathogen susceptibility in amphibians (Belden and Kiesecker, 2005). Biotic and abiotic environmental stressors such as overcrowding, acidification, food deprivation, predation, temperature stress and pollution can elicit an increase in corticosterone levels in amphibian larvae (e.g. Belden *et al.*, 2010; Searle *et al.*, 2010; Crespi and Warne, 2013), while UVBR exposure can increase circulating levels of glucocorticoids in some fish species (Markkula *et al.*, 2007). Whether UVBR exposure is capable of elevating glucocorticoid levels in amphibians, however, remains largely unexplored. In the only study to date, *Rana cascadae* larvae reared under ambient solar UVBR levels showed a slight, but non-significant, increase in corticosterone levels after 7 and 42 days of exposure (Belden *et al.*, 2003). Further work is needed to determine if solar UVBR can induce a chronic stress response in other amphibian species, and if this may contribute to immunosuppression leading to increased pathogen susceptibility.

In amphibians, thyroid hormones (THs), along with glucocorticoids, play a major role in the timing and control of tissue development and metamorphosis (Denver, 2009; Croteau *et al.*, 2010). Changes in the levels of these hormones in response to environmental stressors can, therefore, influence growth rates, tissue development and metamorphic timing (Denver, 2009). UVBR exposure has been shown to impede larval development and reduce deiodinase 2 expression in some tissues of *Rana pipiens* tadpoles and (Croteau *et al.*, 2009) suggesting that UVBR exposure may impair development via its influence on the thyroid axis. Environmental factors that influence neurohormonal pathways are likely to influence a number of physiological systems, including the transition from the larval- to adult-type immune system (Rollins-Smith and Smits, 2004). Indeed, maturation of the larval immune system is highly dependent on both TH and glucocorticoid levels (Rollins-Smith and Blair, 1990; Rollins-Smith, 1998). Conceivably then, larval UVBR exposure may disrupt both the glucocorticoid- and TH-signalling pathways to impair immune system development. While this question is yet to be explicitly explored, Ceccato *et al.* (2016) showed that juvenile frogs reared as larvae under moderate levels of UVBR, had lower levels of circulating leucocytes and reduced antigen swelling responses suggesting a delayed transition from larval to adult phenotype. Recently metamorphosed frogs are at greater risk from pathogens because of the relative immaturity of the immune system (Rollins-Smith, 2017), so there is potential for early UVBR exposure to compound this risk by delaying the development of the adult-type immune system. The role that the glucocorticoid or TH axes played in these responses remains to be determined.

#### Energy production and supply to the immune system

Immune responses and resistance to pathogens and parasites are physiologically demanding (Demas *et al.*, 1997; Martin *et al.*, 2003; Hawley and Altizer, 2011). Energetic costs associated with UVBR exposure (including UVBR damage repair or avoidance mechanisms) may result in trade-offs, reducing an animal’s capacity to mount an effective immune response. Consistent with this view, studies of amphibian larvae have shown that energetic costs associated with repair or avoidance of UVBR damage may be significant, compromising larval growth and foraging opportunities (van de Mortel and Buttemer, 1998; Alton *et al.*, 2010, 2011). Direct UVBR-induced damage to biomolecules within mitochondria (e.g. ROS damage) may also compromise energy production (Cramp *et al.*, 2014). Studies of amphibian larvae have also shown that increased cutaneous melanin production in response to UVBR (Bancroft, 2007) may limit the amount of energy available for growth and, potentially, immune function as well—a view supported by studies of damselflies showing a trade-off between melanin production and immune function (Debecker *et al.*, 2015). Whether this trade-off in energy investment influences the outcome of a pathogen challenge, however, remains to be determined.

#### Carryover effects from early life experiences

The effects of UVBR on amphibian immune function may not manifest immediately, which may mask the potential risk from this stressor. Juvenile amphibian immune function can be significantly influenced by environmental stressors experienced during the larval period, including pond drying and dietary stress (Gervasi and Foufopoulos, 2008; Venesky *et al.*, 2012; Krynak *et al.*, 2015); however, relatively little is known about how early UVBR exposure might influence long-term immune function in amphibians. UVBR has been linked to long-term effects on immune function though direct DNA damage (mutations) as well as through epigenetic processes in mammals (e.g. Gronniger *et al.*, 2010; Shen *et al.*, 2017). For instance, the development of skin cancer in humans follows UVBR-induced immunosuppression that can occur many decades after the causative exposure (Elmets *et al.*, 2014). Early development is a particularly vulnerable stage for mutagenic and epigenetic modifications, since it is a period of rapid cell replication (Perera and Herbstman, 2011). Given that early life stages are most likely to experience UVBR, our group has been exploring the capacity for early UVBR exposure to have a delayed effect on immune function in amphibians. We have found that juvenile frogs, exposed to elevated UVBR as larvae, have reduced antigen responses, reduced white blood cell counts (Ceccato *et al.*, 2016) and are more susceptible to *Bd* than those exposed to low/no UVBR as larvae. These findings indicate that early developmental exposure to UVBR has long-term ramifications for amphibian immune function and disease susceptibility. Further work is required to understand the mechanistic basis for the latent effects of UVBR exposure on amphibians, and how UVBR dose, intensity and the timing of exposure can influence latent immunosuppression.

#### Interactions with other environmental stressors

Like most animals, amphibian immune function can be affected, directly and indirectly, by a range of environmental stressors including temperature (Raffel *et al.*, 2006), diet quality (Venesky *et al.*, 2012), aquatic pH (Krynak *et al.*, 2015), predator stress (e.g. Groner *et al.*, 2013), social stress (Groner *et al.*, 2014; Burraco and Gomez-Mestre, 2016; Aspbury *et al.*, 2017) and chemical contaminants (e.g. Cary *et al.*, 2014; Sifkarovski *et al.*, 2014). UVBR can interact significantly with many of these and other environmental stressors to alter their effects on amphibian physiology (Bancroft *et al.*, 2008; Alton and Franklin, 2017). One particularly well-studied interaction is between UVBR and environmental temperature. In amphibians, exposure to elevated UVBR exacerbates the negative effects of low temperatures on survival, growth and performance (Grant and Licht, 1995; Broomhall *et al.*, 2000; van Uitregt *et al.*, 2007). UVBR compounds the effects of low temperatures by inducing DNA damage, which is slow to be repaired because DNA repair processes are thermally sensitive (Lamare *et al.*, 2006). We have recently found that enzymatic DNA repair rates are ~50% slower at 20°C than they are at 30°C in early *Limnodynastes peronii* larvae (unpublished data). The accumulation of UVBR-associated DNA damage underpins the immunosuppressive nature of UVBR in mammals (Kripke, 1981), but it is unclear if the same is true for amphibians. Conceivably, cool temperatures may impair DNA repair mechanisms, resulting in the accumulation of UVBR-associated damage that triggers immunosuppression. If this is the case, thermal effects on UVBR-associated DNA repair rates may explain some disease-related amphibian declines at high elevation where UVBR levels are naturally high and temperatures are low (van Uitregt *et al.*, 2007). However, further work is needed to build the mechanistic link between UVBR exposure, low temperature and disease susceptibility across species and populations.

## Conclusions and future directions

In this review, we have explored the potential for one changing environmental variable, UVBR, to influence amphibian fitness through its capacity to exert direct and indirect effects on immune function. While relatively little is known about how UVBR can influence amphibian immune function, a large body of literature from studies of other taxa strongly supports the idea that UVBR exposure may increase the susceptibility of amphibians to disease. The potential immunosuppressive effects of UVBR, however, have been largely overlooked in studies investigating the cause(s) of disease-related amphibian population declines. Understanding the role that elevated UVBR levels may have played and may continue to play, in the emergence or exacerbation of amphibian diseases is, therefore, important.

Anthropogenic increases in solar UVBR exposure correlate positively with patterns of enigmatic amphibian decline in many parts of the world (Kiesecker *et al.*, 2001; Middleton *et al.*, 2001). While a novel pathogen (*Bd*) was subsequently shown to be the proximate cause of many of these declines, the possibility that increased UVBR may have contributed to the emergence and epidemiology of the disease has remained largely unexplored. Elevated UVBR has been largely neglected from major environmental models of *Bd*-related declines because of a lack of data on natural UVBR levels in the amphibian–pathogen microenvironment. UVBR levels are difficult to accurately quantify and can vary enormously, over both space and time, as a consequence of solar distance and angle, vegetation cover, cloud thickness and levels of dissolved organic matter in water (Xenopoulos and Schindler, 2001). UVBR levels also co-vary with other changing environmental factors such as temperature, cloud cover and rainfall patterns. Temperature and rainfall significantly influence amphibian behaviour as well as incident UVBR levels, so disentangling the potential negative effects of increased UVBR exposure from the effects of other, co-varying environmental factors, has been, and remains, a complex challenge.

Satellite-based monitoring of global ozone levels and incident UVBR provide a relatively coarse measure of exposure risk and can be used to monitor changes in UVBR levels. Satellite-based UVR data have been used in a small number of studies to model *Bd* infection patterns over a relatively broad spatial scale (the Iberian Peninsula) (Walker *et al.*, 2010; Ortiz-Santaliestra *et al.*, 2011). While the data indicated that *Bd* presence in the study populations was not predicted by any one environmental factor, the conditional prevalence of infection was weakly, negatively correlated with solar radiation levels (Walker *et al.*, 2010). However, the authors stress caution in the interpretation of these findings, mainly because of the incongruity of scale between the coarse climatic statistics used in these studies and the actual microenvironments occupied by both the hosts and the pathogen (Walker *et al.*, 2010). In order to understand if/how UVBR may impact amphibian health and disease processes and to mitigate potential risks for highly threatened species or insurance populations, we need a better understanding of how amphibians interact with UVBR in their environment. Future studies need to consider realistic measures of UVBR in the immediate environment where animals are most likely to be exposed and consider both spatial and temporal variation in UVBR exposure patterns. Likewise, further work is needed to understand how amphibians behaviourally influence individual exposure histories through their choice of oviposition site (e.g. Palen *et al.*, 2005; Thurman *et al.*, 2014) and/or through avoidance or thermoregulatory activities.

Following on from this, the question of ‘how much UVBR is too much’ is key to assessing whether anthropogenic increases in UVBR have contributed to some disease-associated amphibian declines. Amphibians, as a group, occupy a diverse array of environments and may experience a wide range of UVBR levels: how much UVBR is too much is likely to be highly species, population and life-stage specific. For instance, species or populations naturally living in high altitude, open canopy and/or low DOC environments typically experience higher levels of UVBR than fossorial, or fully nocturnal species. Moreover, exposure to UVBR only becomes problematic when an animal’s capacity to respond to, or repair, induced damage is exceeded. While amphibians employ a range of defences against UVBR, their capacity to invoke these can differ enormously across species, populations and life history stages (van de Mortel and Buttemer, 1998; Belden *et al.*, 2003; Palen *et al.*, 2005). Therefore, the threshold level for UVBR exposure above which repair/avoidance strategies are ineffective is likely to be highly context (species, life history stage, population, etc.) specific. Future work is needed to assess whether UVBR exposure thresholds exist for immunosuppression. Thresholds for UVBR-associated immunosuppression are likely to be different for embryos vs larvae and adults and differ across species and/or populations. Understanding the factors that influence the performance of morphological and molecular defence mechanisms, and the plasticity within repair and defence mechanisms to contend with diverse and variable UVBR levels is needed. Likewise, the capacity for larvae to detect and behaviourally modulate their exposure to UVBR needs to be more widely explored.

Ecoimmunological studies often measure immune system responses to environmental stimuli as a proxy for individual fitness (Downs and Stewart, 2014). However, increasingly, studies that employ single immune assays, like white blood cell counts or lectin-induced swelling assays, have been criticized for being overly simplistic or overinterpreted due to the challenge of linking immune measures to actual pathogen susceptibility, resistance or recovery (Hawley and Altizer, 2011). There is also often a lack of correlation among immune assays within single individuals or species (Hawley and Altizer, 2011). Attempts to link host susceptibility to pathogens with responses to non-pathogenic immune assays are hampered by a lack of understanding about which immune parameters will best predict pathogen susceptibility. Moreover, different pathogens may differentially interact with the immune system, and so markers that may predict outcomes for one pathogen, may bear no relevance to infection outcomes for another pathogen. Consequently, it is important that future studies of UVBR on amphibian immune function examine a suite of traits that reflect the diverse ways that the immune system may to respond to a challenge, both within and across life stages, including by directly measuring pathogen susceptibility, infection intensity and immune responses to antigenic challenges.

Immune function is a key individual-level trait that influences populations because it directly affects the survival outcome of a pathogen challenge (Downs and Stewart, 2014). However, within a community, variability in host immune function can promote selection and the evolution of resistance to infection (Lazzaro and Little, 2009). Variability in the responses of immune system components to the environment can promote polymorphism within a population; environmental heterogeneity can then maintain genetic variation in immunity by promoting alternative phenotypes over space and time (Lazzaro and Little, 2009). Given that UVBR exposure history likely differs significantly within and between populations, UVBR may differentially influence selection pressure on amphibian immune function genes, which in turn may shape differences in disease prevalence and infection outcome. Relatively fast-paced changes in UVBR levels may have placed additional pressure on populations, as the rate and magnitude of change could have exceeded the capacity for evolutionary mechanisms to maintain genetic heterogeneity in immune traits. This may have rendered some amphibian populations or species more susceptible to novel pathogens and contributed to the catastrophic declines that followed. Analysis of the way that UVBR can shape genetic heterogeneity within and between species and populations would inform our understanding of how UVBR may influence the evolution of disease resistant or susceptible phenotypes.

Historically, the primary role of the immune system has been regarded as the detection and elimination of pathogens (i.e. ‘resistance’). While in this review we have focused on the potential for UVBR to disrupt amphibian resistance pathways, tolerance of pathogens is increasingly recognised as an important immune strategy for managing host–pathogen interactions (Medzhitov *et al.*, 2012). Tolerance can be defined as responses that limit the negative effects of the infection on the host without influencing the pathogen burden. Resistance-based immune responses are energetically costly and often damaging to the host; tolerance of a pathogen may allow hosts to maximize their fitness by preventing costly immunopathological responses (Medzhitov *et al.*, 2012). The concept of tolerance is relatively new to ecoimmunology and the mechanisms underpinning it are poorly understood. Conceivably though, UVBR exposure may shape disease dynamics within populations by reducing the capacity of hosts to tolerate otherwise manageable microbial interactions. Conversely, UVBR has been shown to impair some resistance pathways, increasing pathogen/allergen tolerance, termed ‘photo-tolerance’ (Spellman *et al.*, 1984; de Gruijl, 2008). While pathogen tolerance may be an effective strategy to minimize the negative effects of a pathogen on an individual, tolerant hosts can also serve as highly infectious vectors of disease (Medzhitov *et al.*, 2012). This has potentially significant implications for the spread of pathogens within and between populations. Several amphibian species, including the American bullfrog (*Lithobates catesbeianus*) and the clicking froglet (*Crinnia signifera*), have been identified as highly virulent vectors of *Bd* capable of harbouring and spreading the fungus to more susceptible species in their environments (Schloegel *et al.*, 2012; Brannelly *et al.*, 2018). Further work is required to determine if UVBR exposure regimes can influence pathogen tolerance in amphibians and if this may contribute to inter and intraspecific differences in susceptibility to pathogens like *Bd*.

Global UVBR levels have increased by between 2 and 6% since the middle of the last century (Lemus-Deschamps and Makin, 2012) and are expected to remain significantly elevated for much of this century (Ball *et al.*, 2018; Montzka *et al.*, 2018). Elevated UVBR levels correlate broadly with patterns of disease-related amphibian biodiversity loss, particularly at high altitude (Kiesecker *et al.*, 2001; Middleton *et al.*, 2001), yet potential causal links between UVBR exposure and amphibian disease remain relatively unexplored. UVBR is a powerful immunosuppressant in other animals and likely affects amphibians in the same way. However, further work is needed to establish the mechanistic link between UVBR, immune function and disease susceptibility in amphibians. By taking a physiological approach to understanding the mechanisms through which the environment shapes amphibian immune function, we can better predict how environmental change is likely to influence disease dynamics with and between species and populations. Physiological studies are uniquely positioned in this regard, because environmental effects on physiology can be experimentally tested. Therefore, unlike correlative studies, empirical physiological studies can provide the compelling evidence needed to establish direct cause-and-effect (Carey, 2005; Cooke *et al.*, 2013). The diversity of ways in which the environment can shape population and species persistence via impacts on individual physiology, highlights the importance of taking a holistic and integrative approach to address ecoimmunological problems in a rapidly changing world. We have identified a number of direct and indirect pathways through which elevated UVBR could shape individual immune function and influence disease processes in amphibians, which we hope may guide future research in this field. Currently, our understanding of how UVBR and disease influence amphibians is restricted to a relatively small number of ‘model’ species. This represents a critical knowledge gap which may bias our responses to population declines in under-represented species. There remains an urgent need for fundamental data on how the environment shapes amphibian immune responses across orders, species, populations and life stages, and how this influences their capacity to resist pathogen infections.
